# Emodin-Induced Oxidative Inhibition of Mitochondrial Function Assists BiP/IRE1*α*/CHOP Signaling-Mediated ER-Related Apoptosis

**DOI:** 10.1155/2021/8865813

**Published:** 2021-04-22

**Authors:** Li-zhen Qiu, Lan-xin Yue, Yu-hao Ni, Wei Zhou, Cong-shu Huang, Hui-fang Deng, Ning-ning Wang, Hong Liu, Xian Liu, Yong-qiang Zhou, Cheng-rong Xiao, Yu-guang Wang, Yue Gao

**Affiliations:** ^1^Department of Pharmaceutical Sciences, Beijing Institute of Radiation Medicine, Beijing 100850, China; ^2^Tianjin University of Traditional Chinese Medicine, Tianjin 301617, China; ^3^School of Traditional Chinese Medicine, Guangdong Pharmaceutical University, Guangzhou 510006, China

## Abstract

Cassiae Semen is a widely used herbal medicine and a popular edible variety in many dietary or health beverage. Emerging evidence disclosed that improper administration of Cassiae Semen could induce obvious liver injury, which is possibly attributed to emodin, one of the bioactive anthraquinone compounds in Cassiae Semen, which caused hepatotoxicity, but the underlying mechanisms are not completely understood. Hence, the present study firstly explored the possible role of oxidative stress-mediated mitochondrial dysfunction and ER stress in emodin-cause apoptosis of L02 cells, aiming to elaborate possible toxic mechanisms involved in emodin-induced hepatotoxicity. Our results showed that emodin-induced ROS activated ER stress and the UPR via the BiP/IRE1*α*/CHOP signaling pathway, followed by ER Ca^2+^ release and cytoplasmic Ca^2+^ overloading. At the same time, emodin-caused redox imbalance increased mtROS while decreased MMP and mitochondrial function, resulting in the leaks of mitochondrial-related proapoptotic factors. Interestingly, blocking Ca^2+^ release from ER by 2-APB could inhibit emodin-induced apoptosis of L02, but the restored mitochondrial function did not reduce the apoptosis rates of emodin-treated cells. Besides, tunicamycin (TM) and doxorubicin (DOX) were used to activate ER stress and mitochondrial injury at a dosage where obvious apoptosis was not observed, respectively. We found that cotreatment with TM and DOX significantly induced apoptosis of L02 cells. Thus, all the results indicated that emodin-induced excessive ROS generation and redox imbalance promoted apoptosis, which was mainly associated with BiP/IRE1*α*/CHOP signaling-mediated ER stress and would be enhanced by oxidative stress-mediated mitochondrial dysfunction. Altogether, this finding has implicated that redox imbalance-mediated ER stress could be an alternative target for the treatment of Cassiae Semen or other medicine-food homologous varieties containing emodin-induced liver injury.

## 1. Introduction

Cassiae Semen, the dried and ripe seed of *Cassia obtusifolia L.* or *Cassia tora L.*, is a well-known Chinese herbal medicine and has been used for more than 2000 years in China and other East Asian countries, thanks to its remarkable efficacy for hepatoprotection, lowering blood pressure, improving eyesight, and antioxidation [1]. Besides, as a popular edible substance and a common ingredient in many slimming health foods and beverages, Cassiae Semen has displayed excellent performance in the control of blood lipid and body weight [2]. However, this medicine-food homologous variety has been demonstrated to exhibit obvious hepatoxicity that can eventually result in liver damage and cholestasis [3]. The safety of Cassiae Semen has therefore become a global health concern.

In recent years, there is a growing body of evidence that emodin, an anthraquinone compound extracted from Cassiae Semen, is not only a major bioactive substance but also the main hepatoxic substance of this edible herbal medicine [4]. As documented in previous researches, emodin could not only attenuate CCl_4_-, APAP-, and ethanol-induced hepatotoxicity of hepatocytes by inhibiting oxidative stress or lipid peroxidation but also ameliorate LPS-caused fulminant hepatic failure by suppressing immune responses [5]. Given such hepatoprotective activity, emodin was once considered a promising medicine for different liver diseases. But the latest studies showed that emodin (more than 30 *μ*M) dose-dependently caused significant disturbance of fatty acid metabolism and apoptosis in human hepatocytes [6, 7]. Pharmacokinetics studies confirmed that emodin was the critical hepatotoxic component of other medicines containing emodin [8]. Nevertheless, the mechanisms by which emodin induces hepatotoxicity are not yet fully understood.

Reactive oxygen species- (ROS-) initiated perturbation of redox homeostasis and the subsequent oxidative stress always participate in xenobiotic-induced hepatoxicity. Generally speaking, excessive accumulation of intracellular ROS could cause oxidative injuries to biomacromolecules and organelles, mostly resulting in apoptotic cell death [8]. Once ROS causes a decrease in the mitochondrial membrane potential and an increase in mitochondrial membrane permeability, mitochondrial proapoptotic factors will be released to the cytoplasm and trigger the mitochondrial-dependent apoptosis [8]. What is worthy of note is that elevated intracellular ROS generation directly induces the endoplasmic reticulum (ER) stress, which could in turn activate the ER-related apoptotic cell death that is characterized by the calcium release from the ER and the activation of Caspase-12 [9, 10]. More importantly, mitochondrion and the ER coordinate the oxidative stress responses and play an important role in the initiation of apoptosis.

Previous articles have reported that emodin (50 *μ*M) did have an impact on mitochondrial oxidative phosphorylation in hepatocytes and triggered ROS-mediated ER stress in human T cells [11], suggesting that emodin-caused hepatotoxicity would be associated with ROS-induced mitochondrial dysfunction and ER stress. Unfortunately, no report is currently available that emodin induces ROS generation and the consequent ER stress- and/or mitochondrial-related apoptosis in hepatocytes. Instead, emodin (10 *μ*M) could inhibit oxidative stress generated by arachidonic acid plus iron [12]. Thus, the role of ROS-mediated mitochondrial dysfunction, ER stress, and related apoptotic cell death in hepatotoxicity caused by emodin needs to be explored.

In the present study, to determine the toxicity effects of emodin on the liver, the cytotoxicity and redox status of emodin-treated human hepatocytes were examined before the mitochondrial function, ER stress, and toxic mechanisms involved in presenting a possible mode of action of emodin-induced hepatotoxicity were investigated. Furthermore, the role of reduced mitochondrial function in emodin-induced ER-related apoptosis was also discussed.

## 2. Methods and Materials

### 2.1. Cell Culture and Reagents

The normal human hepatocyte cell line (L02) was purchased from the Type Culture Collection of the Chinese Academy of Sciences, Shanghai, China. The L02 cells were cultured in complete RPMI-1640 medium (Gibco, Thermo Fisher Scientific, USA) containing 10% fetal bovine serum (FBS, 11011-861, EVERY GREEN) and 1% penicillin-streptomycin (10 kU/mL-10 mg/mL, FG101-01, Transgene, China) and maintained in an incubator with 5% CO_2_ at 37°C. The culture medium was renewed every two days, and cells were passaged at 80% confluence using 0.25% trypsin-EDTA. Emodin was purchased from EFEBIO Company (E054323, 98% (HPLC), China). (2-(2,2,6,6-Tetramethylpiperidin-1-oxyl-4-ylamino)-2-oxoethyl) triphenylphosphonium chloride (MitoTEMPO or MT, SML0737, ≥98% (HPLC)) and 2-aminoethyl diphenylborinate (2-APB, #9754, 97% (HPLC)) were from Sigma-Aldrich (China). N-Acetyl-L-cysteine was acquired from Beyotime Biotechnology (NAC, S0077, >99%, China). Tunicamycin (TM) and doxorubicin (DOX) were purchased from Solarbio Life Science (T8480 and D8740, China), and their purities were both more than 98%.

### 2.2. Cell Viability Assay

Cell viability was determined using the Cell Counting Kit-8 assay (CCK-8, Dojindo, Japan) according to the manufacturer's instructions. Cells were seeded in a 96-well plate and treated with emodin (0, 6.25, 12.5, 25, and 50 *μ*M) for 24 h. At the end of treatment, cells were washed with PBS buffer and incubated with RPMI-1640 medium containing 10% CCK-8 solution for 2 h in an incubator with 5% CO_2_ at 37°C. Sequentially, the absorbance of each well was measured with a microplate reader at 450 nm (Multiskan MK3, Thermo Fisher Scientific, USA). Cell viabilities for treated groups were presented as percentages of that of the control.

### 2.3. Determination of Cell Apoptosis Rate

An Annexin V-FITC/PI apoptosis detection kit (C1062L, Beyotime, China) was used to detect the apoptosis of L02 cells according to the manufacturer's instructions. The L02 cells (3 × 10^5^) were seeded in 60 mm plates and treated with emodin (25 *μ*M, with or without MT (5 *μ*M), 2-APB (20 *μ*M)), TM (62.5 nM), and DOX (125 nM). After treatment, the cells were collected into 15 mL centrifugal tubes and washed with warm PBS. Then, cells in each group were centrifuged for 5 min at 1000 rpm at room temperature (RT). Having the supernatant discarded, 195 *μ*L 1x binding buffer was used to resuspend cells in 1.5 mL centrifugal tubes, followed by 5 *μ*L Annexin V-FITC and 10 *μ*L PI for double staining. Soon after incubation in the dark at room temperature (RT) for 10 min, the cell suspensions were submitted to a flow cytometer (FACSCalibur, Becton Dickinson, USA) to determine apoptosis rates at an excitation wavelength of 488 nm.

### 2.4. Measurement of Intracellular ROS

The L02 cells were placed into 35 mm plates at a density of 1 × 10^5^ cells. The next day, the cells were treated with 6.25, 12.5, 25, and 50 *μ*M of emodin with or without 5 mM NAC for 24 h. After being washed with warm PBS, the cells were incubated with 1 mL of serum-free medium containing 10 *μ*M of 2,7-dichlorofluorescein diacetate (DCFH-DA) (C1300-1, Applygen Technologies, China). After 30 min of incubation at 37°C, the cells were washed twice by PBS before the images were captured by one inverted fluorescence microscope (Ti2, Nikon, Japan). Fluorescence images were analyzed using ImageJ software, while fluorescence intensities in treated groups were expressed by normalized values to that of the control.

### 2.5. Confocal Microscopy

Cells placed into 20 mm glass-bottomed culture dishes were treated with emodin (25 *μ*M) and with or without MitoTEMPO (5 *μ*M) or 2-APB (20 *μ*M) when they grew to 50-70% confluence. After 24 h treatment, the lipophilic cationic fluorescent dye JC-1 (KeyGEN, Nanjing, China) was used to detect the changes in the mitochondrial membrane potential (MMP), and the MitoSOX™ Red mitochondrial superoxide indicator (M36008, Thermo Fisher Scientific, USA) was applied to determine the mitochondrial superoxide anion. All the procedures were performed based on the manufacturer's protocol. For JC-1, fresh staining solution JC-1 (1x) was prepared immediately with a stock solution of JC-1 (200x) diluted in ultrapure water and JC-1 staining buffer (5x) before use. The cells were incubated with 1 mL of JC-1 (1x) for 20 min at 37°C, then washed twice with cold PBS, and examined immediately on a Confocal Laser Scanning Microscope (Leica TCS-SP2 confocal microscope). Fluorescence was excited at 514 nm and measured between 514 and 529 nm (green) and at 585-590 nm (red). For MitoSOX detection, cells were incubated in a warm medium containing 5 *μ*M MitoSOX reagent. After 10 min of incubation at 37°C, these cells were washed with PBS and analyzed by the same confocal microscope but at (Ex/Em) 510/580 nm. All image acquisitions and analyses were performed using Volocity demo software. The intensity of the laser beam and the photodetector sensitivity were kept constant to compare the relative fluorescence intensities between these groups.

### 2.6. Detection of the Level of Cytoplasmic Calcium (Ca^2+^)

Fluo-4 AM (F312, Dojindo, Japan) was used to detect changes of calcium ions (Ca^2+^) in the cytoplasm. The cells were seeded in glass culture dishes at a density of 2.5 × 10^4^/mL. After 24 h treatment with emodin (25 *μ*M) with or without MitoTEMPO (5 *μ*M) or 2-APB (20 *μ*M), cells were stained with the 1 *μ*M Fluo-4 AM for 40 min according to the manufacturer's descriptions. The extra dye was removed by a three-time wash with HBSS. The concentration of cytoplasmic Ca^2+^ was detected with a confocal microscope, the excitation wavelength was 494 nm, and the emission wavelength was 516 nm.

### 2.7. Western Blotting Analysis

After emodin treatment, the cells were washed and lysed with RIPA buffer (C1053+, Applygen Technologies) and protease inhibitor cocktail (P1265, Applygen Technologies) to collect proteins. A bicinchoninic acid (BCA) protein assay kit (P1511, Applygen Technologies) was used to quantify the concentrations of whole-cell proteins of each group. The proteins were separated using a sodium dodecyl sulfate-polyacrylamide gel electrophoresis (SDS-PAGE) and then transferred to PVDF membranes (IPVH00010, Millipore, Germany), subsequently blocked in 5% milk diluted in tris-buffered saline solution with 0.05% Tween 20 (TBST) for 2 h at RT and incubated with primary antibody at 4°C overnight. The membranes were washed with TBST for 3 × 10 min and incubated with secondary antibodies at RT for 1 h. After another three-time wash, the expressions of proteins were detected by enhanced chemiluminescence (ECL) according to the manufacturer's instructions (WBKLS0500, Millipore). The band intensity of each protein was quantified using ImageJ software. *β*-Actin was used as a loading control to normalize the protein expression. Information for all indicated antibodies used in our study could be found in supplementary materials (Table S1).

### 2.8. Seahorse XF Cell Mito Stress Test

Oxygen Consumption Rates (OCRs) and Extracellular Acidification Rates (ECARs) were both detected in real time using the Mito Stress Test Kit (103015-100, Agilent) and carried out with a Seahorse XFe96 Analyzer (Seahorse Bioscience, Agilent, Texas, USA) according to the manufacturer's instructions. Briefly, the cells were seeded in a Seahorse XF 96-well microplate (8000 cells/well, 101085-004, Agilent). After drug treatment, cells were incubated at 37°C with 5% CO_2_ for 24 h. Then, the cells were washed and incubated in Seahorse XF assay medium (103575-100, Agilent) supplemented with 2 mM Glutamax (103579-100, Agilent), 1 mM sodium pyruvate (103578-100, Agilent), and 25 mM glucose (103577-100, Agilent) at 37°C for 1 h without CO_2_. Base OCR values were measured, followed by sequential drug injection including oligomycin (2 *μ*M), carbonyl cyanide-p-trifluoromethoxy-phenylhydrazone (FCCP, 0.5 *μ*M), and rotenone/antimycin A (1 *μ*M), respectively, and the detection of OCR values, which would stand for the ATP production (proton leak), maximal respiration, and spare capacity in each group. Finally, cell numbers were counted by DAPI staining after the Mito Stress Test XF metabolic assay using a Scan High-Content System (Thermo Fisher Scientific, Massachusetts, USA). The mitochondrial respiratory capacity of each group was normalized to total cell numbers and presented as pmol/min/10,000 cells.

### 2.9. Statistical Analysis

All results were presented as mean ± standard deviation (SD) and generated from at least three independent experiments. One-way ANOVA followed by the LSD post hoc test for multiple comparisons was performed for statistical analysis. Only at *p* < 0.05 was considered significant.

## 3. Results

### 3.1. Elevated Intracellular ROS Mediated Emodin-Induced Cytotoxicity on Human Hepatocytes

**Figure 1:**
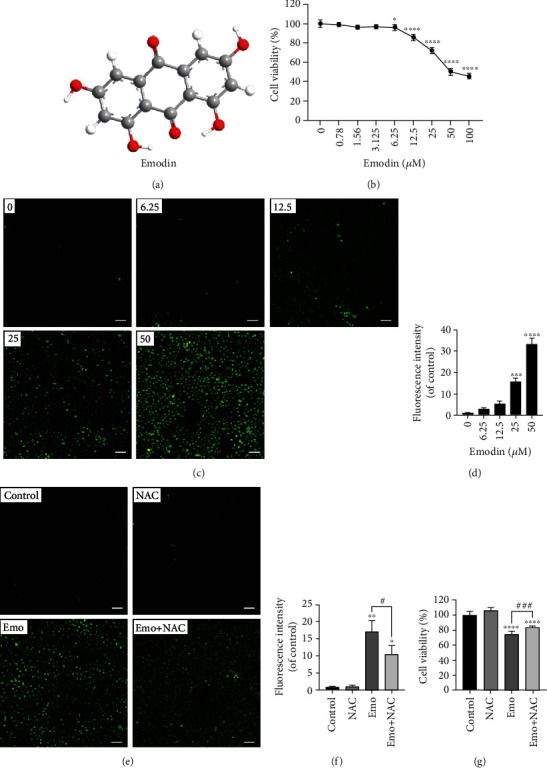
Emodin inhibited the viability of L02 cells via enhancing intracellular ROS. (a) Chemical structure of emodin. (b) Cell viability of L02 cells after 24 h emodin treatment was detected using the CCK-8 assay. (c, d) Intracellular ROS levels in L02 induced by emodin (×200), scale bar: 100 *μ*m. (e, f) Intracellular ROS levels in L02 treated with NAC, emodin, or NAC+emodin (×200), scale bar: 100 *μ*m. (g) Cell viability of L02 cells treated with NAC, emodin, or NAC+emodin. ^∗^*p* < 0.05, ^∗∗^*p* < 0.01, ^∗∗∗^*p* < 0.001, and ^∗∗∗∗^*p* < 0.0001 vs. the control groups; ^#^*p* < 0.05, ^###^*p* < 0.001 vs. the emo groups.

To determine the cytotoxicity of emodin on human hepatocytes, we exposed L02 cells to various emodin of concentrations (0-100 *μ*M, Figure 1(a)) for 24 h. As shown in Figure 1(b), cell viability was reduced significantly by emodin in a dose-dependent manner. It was found that 25 *μ*M of emodin decreased L02 cell viability to 72.10 ± 2.81%. Figures 1(c) and 1(d) showed that emodin dose-dependently increased the intracellular levels of ROS. When L02 cells were cotreated with NAC (an inhibitor of ROS) and emodin (25 *μ*M), intracellular ROS was significantly alleviated (Figures 1(e) and 1(f)). In addition, the cell viability in the NAC and the emodin cotreatment group was evidently increased compared with the emodin group (Figure 1(g)). The results indicated that elevated intracellular ROS promoted emodin-induced cytotoxicity on human hepatocytes.

### 3.2. Emodin Caused ER Stress and Ca^2+^ Overloading through the BiP/IRE1*α*/CHOP Signaling Pathway

**Figure 2:**
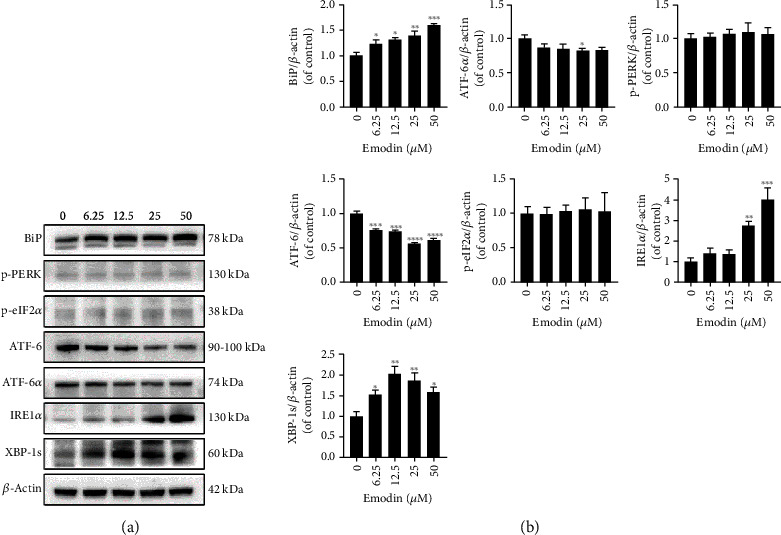
Emodin caused the activation of the BiP/IRE1*α*/CHOP signaling pathway. (a) The protein levels of BiP, p-RERK, p-eIF2*α*, ATF-6, ATF-6*α*, IREI*α*, and XBP-1s in L02 were measured by western blotting assay. (b) The quantitative analysis of the indicated proteins. ^∗^*p* < 0.05, ^∗∗^*p* < 0.01, ^∗∗∗^*p* < 0.001, and ^∗∗∗∗^*p* < 0.0001 vs. the control groups.

**Figure 3:**
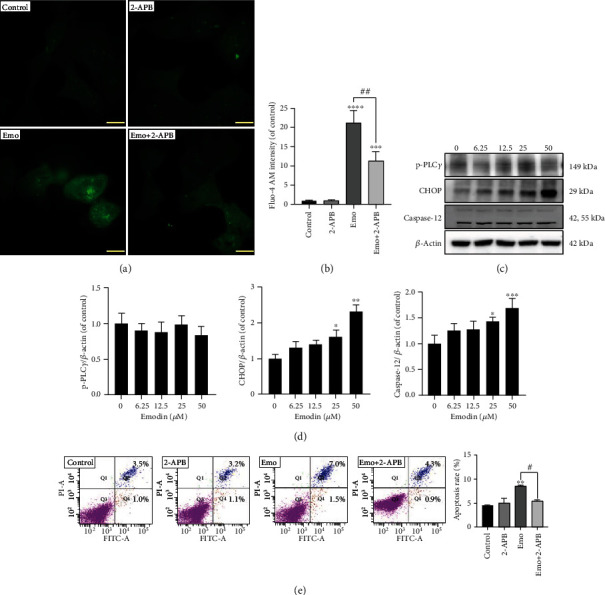
Apoptosis induced by emodin was associated with ER stress-triggered Ca^2+^ release. (a, b) The intracellular concentration of Ca^2+^. Scale bar: 47 *μ*m. (c, d) The protein levels of p-PLC*γ*, CHOP, and Caspase-12 in L02 were detected by western blotting assay. (e) Emodin-induced apoptosis of L02 cells cotreated with or without 2-APB was analyzed using FACS analysis. ^∗^*p* < 0.05, ^∗∗^*p* < 0.01, ^∗∗∗^*p* < 0.001, and ^∗∗∗∗^*p* < 0.0001 vs. the control groups; ^#^*p* < 0.05, ^##^*p* < 0.01 vs. the emo groups.

To evaluate the effects of emodin on the ER, we detected the levels of ER stress-related proteins in cells treated with emodin using western blotting assay. As shown in Figure 2, compared with the control group, the expressions of BiP, IREI*α*, and XBP-1s were markedly increased, while those of ATF-6 and ATF-6*α* were distinctly decreased in the emodin groups in a dose-dependent manner. However, the protein bands of p-RERK and p-eIF2*α* were not different between the emodin groups and the control group. Previous studies have demonstrated that the intracellular Ca^2+^ concentration homeostasis is mostly maintained by the ER. Hence, we stained the L02 cells with Fluo-4 AM to explore the intracellular Ca^2+^ flux induced by emodin. As shown in Figures 3(a) and 3(b), enhanced cytoplasmic Ca^2+^ loading was observed in the emodin-treated group, which was suppressed by cotreatment with 2-APB, an antagonist of Ca^2+^ release from the ER. Therefore, these results suggested that emodin could cause BiP/IRE1*α*/XBP-1s signaling pathway-mediated ER stress, which eventually induced Ca^2+^ release from the ER and resulted in cytoplasmic Ca^2+^ loading.

### 3.3. Emodin-Induced Apoptosis Was Associated with ER Stress-Triggered Ca^2+^ Release

To further elucidate the role of ER stress-indued Ca^2+^ overloading in the apoptosis induced by emodin, the expressions of p-PLC*γ* and CHOP were detected. As shown in Figures 3(c) and 3(d), the protein levels of both CHOP and Caspase-12 were significantly elevated, especially in the high-dose groups. However, the expression of p-PLC*γ* remained unchanged in the emodin groups, indicating that ER stress promoted Ca^2+^ release through CHOP rather than PLC*γ* signaling. Moreover, cotreatment with 2-APB significantly decreased emodin-induced apoptosis of L02 cells, suggesting Ca^2+^ release from the ER participated in emodin-induced apoptosis (Figure 3(e)). Also, the increase in Caspase-12 expression in emodin-treated cells indicated the critical role of the ER in emodin-induced cytotoxicity (Figures 3(c) and 3(d)). Thus, our results demonstrated that emodin-induced apoptosis was mostly ER stress-related.

### 3.4. Overexpression of Superoxide Anion Decreased MMP and Mitochondrial Function

**Figure 4:**
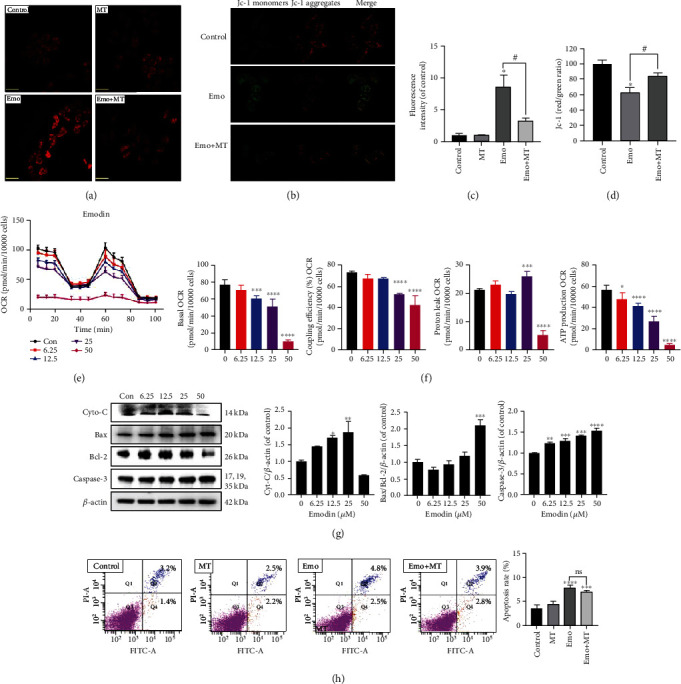
Emodin induced oxidative inhibition of MMP and mitochondrial function. (a, c) The intracellular superoxide anion levels in L02 cells were assessed using MitoSOX. Scale bar: 140 *μ*m. (b, d) The MMP levels in L02 cells were detected using the JC-1 probe. Scale bar: 140 *μ*m. (e) Mitochondrial function in L02 cells treated with emodin (0-50 *μ*M) for 24 h was measured via the Mito Stress Test. (f) Effects of emodin on OCRs of basal, ATP production, proton leak, and coupling efficiency. (g) The protein levels of Cyto-C, Bax/Bcl2, and Caspase-3 in emodin-treated L02 cells were detected using western blotting. (h) Emodin-induced apoptosis of L02 cells cotreated with or without MT was analyzed using FACS analysis. ^∗^*p* < 0.05, ^∗∗^*p* < 0.01, ^∗∗∗^*p* < 0.001, and ^∗∗∗∗^*p* < 0.0001 vs. the control groups; ^#^*p* < 0.05 vs. the emo groups.

There is increasing evidence that apoptosis is also closely related to mitochondrial damage or dysfunction. Thence, to detect the production of mitochondrial superoxide anion and its effects on MMP, the MitoSOX red and JC-1 probes were employed, respectively. Our results showed that emodin upregulated the level of mitochondrial superoxide, which could be relieved by a mitochondrial-targeted antioxidant MT (Figures 4(a) and 4(c)). As shown in Figures 4(b) and 4(d), an obvious decrease in the ratio of red fluorescence to green fluorescence was observed in emodin-treated cells, and this shift could be reversed by MT cotreatment. Then, the Mito Stress Test was performed to evaluate mitochondrial function in cells after emodin treatment of 24 h, and it was found that L02 cells treated with emodin displayed an evident reduction in basal OCRs, ATP production OCRs, and coupling efficiency OCRs (Figures 4(e) and 4(f)). Emodin enhanced the proton leak OCRs at 25 *μ*M, which also indicated significant damage to mitochondrial function (Figure 4(f)). Besides, mitochondrial-related proapoptosis factors were detected. For example, the ratio of Bax/Bcl-2 and the contents of cytochrome C and Caspase-3 were increased in emodin groups (Figure 4(g)). Furthermore, the proportion of apoptosis of L02 cells displayed a marked increase in the emodin group, which could be partly reversed by MT cotreatment, but there was no significant difference (Figure 4(h)). Hence, the results suggested that emodin-induced overexpression of mitochondrial superoxide anion could cause mitochondrial dysfunction, but the apoptosis of emodin-treated cells might not be totally mitochondrial-dependent.

### 3.5. Ca^2+^ Release from ER Participated in Emodin-Induced Mitochondrial Dysfunction

**Figure 5:**
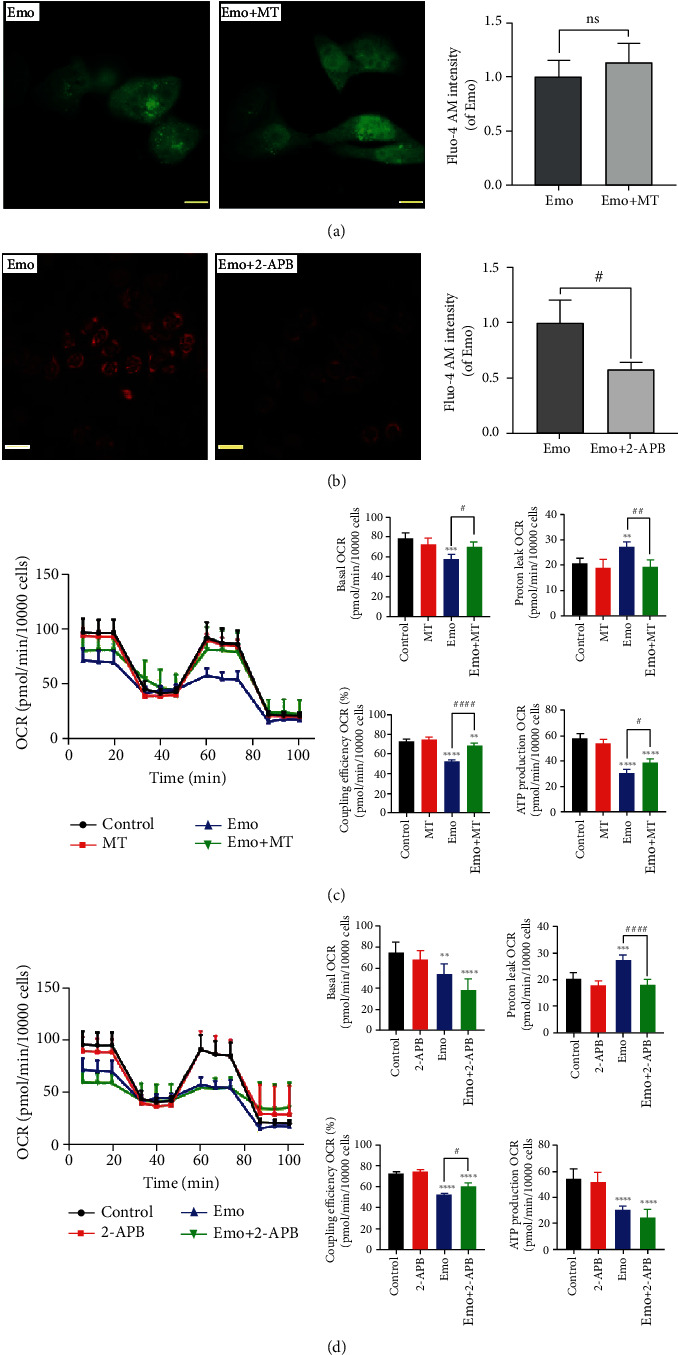
Ca^2+^ release produced by ER stress participated in emodin-induced mitochondrial dysfunction. (a) The intracellular Ca^2+^ flux was stained with the Fluo-4 AM dye. (b) The superoxide anion levels were detected with the MitoSOX probe. (c, d) Mitochondrial functions in the L02 cells treated with MT, emodin, or Emodin+MT, 2-APB, and 2-APB+emodin were obtained by the Cell Mito Stress Test Kit, and the quantification analysis of OCR value of basal, ATP production, proton leak, and coupling efficiency. ^∗^*p* < 0.05, ^∗∗^*p* < 0.01, ^∗∗∗^*p* < 0.001, and ^∗∗∗∗^*p* < 0.0001 vs. the control groups; ^#^*p* < 0.05, ^##^*p* < 0.01, ^####^*p* < 0.0001 vs. the emo groups.

Interestingly, it was found that scavenging of emodin-induced mitochondrial superoxide using MT did not reverse cytoplasmic Ca^2+^ loading compared with the emodin group (Figure 5(a)). However, when Ca^2+^ release from the ER to the cytoplasm was inhibited by 2-APB, emodin (25 *μ*M)-induced mitochondrial superoxide was alleviated (Figure 5(b)). To further elucidate the effects of ER Ca^2+^ release induced by emodin on the mitochondrial function, L02 cells were exposed to emodin with/without MT or 2-APB. As shown in Figure 5(c), MT could restore the reduced function of the mitochondrial respiratory chain induced by emodin. Also, 2-APB cotreatment could markedly reduce emodin-caused proton leak of mitochondria and enhance the coupling efficiency of mitochondrial respiratory chain to some extent in emodin-treated hepatocytes (Figure 5(d)). The results indicated that Ca^2+^ release from the ER participated in emodin-induced mitochondrial dysfunction by disturbing the mitochondrial redox status.

### 3.6. Oxidative-Related Decrease in Mitochondrial Function Participated in ER-Related Apoptosis

**Figure 6:**
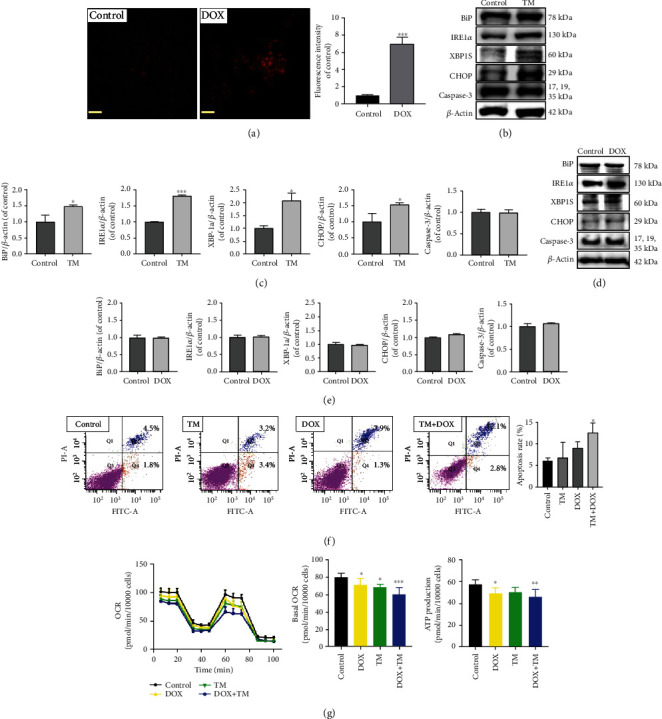
Oxidation-related decrease in mitochondrial function participated in ER stress-related apoptosis. (a) The mtROS levels in L02 cells treated with DOX were measured using the MitoSOX probe. (b–e) The expressions and quantification analysis of BiP, IRE1*α*, CHOP, and Caspase-3 in cells treated with TM and DOX. (f) The apoptosis rates of L02 cells treated with DOX, TM, or DOX+TM were analyzed using FACS analysis. (g) Mitochondrial functions in the L02 cells treated with TM, DOX, or TM + DOX for 24 h, and the effects of these treatments on OCRs of basal and ATP production. ^∗^*p* < 0.05, ^∗∗^*p* < 0.01, and ^∗∗∗^*p* < 0.001 vs. the control groups.

To further explore the relationship between emodin-induced mitochondrial dysfunction and ER stress, DOX and TM were chosen as positive drugs to induce mitochondrion injury and ER stress, respectively. Neither of them caused any significant decrease in cell viability at the indicated concentration (data not shown). Indeed, excessive mitochondrial superoxide anion was detected in L02 cells treated with DOX (125 nM) (Figure 6(a)); TM (62.5 nM) activated the BiP/IRE1*α*/CHOP signaling pathway without increasing Caspase-3 content (Figures 6(b) and 6(c)), but DOX did not induce the activation of this signaling pathway (Figures 6(d) and 6(e)). As shown in Figure 6(f), compared with control, DOX or TM administration alone did not induce significant increases in apoptosis of L02 cells, which also provided more evidence that neither DOX at 125 nM nor TM at 62.5 nM had a significant effect on cell viability. However, coexposure to DOX and TM could significantly elevate the apoptosis rate from 6.3% to 14.9%, suggesting that oxidative stress-mediated mitochondrial injury might be involved in ER stress-related apoptosis. Moreover, mitochondrial respiratory function analysis also demonstrated that TM exacerbated DOX-induced decreases in basal mitochondrial respiratory function and ATP production (Figure 6(g)). Taken together other results described above, it could be concluded that oxidative inhibition of mitochondrial function decreases ATP supply, which might play an important role in ER stress-related cell apoptosis.

## 4. Discussion

As a popular medicine-food homologous variety, Cassiae Semen has been applied in clinical practice or daily cuisine in China and other East Asia countries for two thousand years, due to the excellent pharmacological effects on vision improvement, kidney and liver protection, and blood lipid control [13]. Recently, there have been many reports on its adverse effects caused by improper administration of traditional Chinese medicines or diet containing Cassiae Semen. Such adverse effects as acute liver injury have raised increasing concerns about the safety of this dietary/medicinal plant seed [3]. Emodin, one of the main bioactive compounds in Cassiae Semen, was considered to be able to alleviate inflammation without toxicity [5]. However, the latest evidence showed that emodin could directly induce cytotoxicity on hepatocytes [6], but the related toxic mechanisms were not entirely understood. Hence, our study was the first to confirm that emodin could promote excessive ROS generation and redox imbalance in human hepatocytes, followed by BiP/IRE1*α*/CHOP signaling-mediated ER stress and the unfolded protein response (UPR) and intracellular Ca^2+^ overloading, resulting in ER-related apoptosis but not mitochondrial-dependent apoptosis. Also, oxidative-related mitochondrial dysfunction played an important role in emodin-induced ER-related apoptosis.

Redox equilibrium is of importance to cell survival and function [14]. Abnormal ROS levels and oxidative stress always participate in the following toxic effects induced by internal toxicants or xenobiotic exposure [15, 16]. A previous study reported that emodin treatment promoted ROS content in the rat liver [17], but several studies found that low doses of emodin could inhibit ROS-mediated oxidative stress in HeLa cells and rat macrophages [12, 18]. In conformity with earlier findings related to non-hepatocytes, our results demonstrated that emodin-induced ROS generation was associated with emodin-caused hepatic cytotoxicity. Metabolomic profiling identified obvious emodin-cysteine adducts in the cell culture medium, indicating that excessive consumption of cysteine-related antioxidants, such as GSH, might play a critical role in emodin-induced ROS generation and oxidative stress in hepatocytes [19]. As the main cellular redox hubs, MMP and mitochondrial function in turn were affected by overgenerated superoxide anion free radicals [20]. Obvious reductions in the coupling efficiency of OXHPS and ATP production suggested the depletion of intracellular ATP content. Meanwhile, the increased cytochrome C release, Bax/Bcl-2 ratio, and Caspase-3 expression suggested that emodin-caused hepatocytes' apoptosis would be possibly mitochondrial-dependent [21]. However, we found that MT indeed restored mitochondrial function but did not significantly decrease emodin-induced apoptosis. Therefore, oxidative stress-mediated inhibition of mitochondrial function could not be the core regulatory pathway of emodin-induced apoptosis of human hepatocytes.

Generally, ROS-induced oxidative stress affects the progress of protein folding, maturation, and degradation, all of which are controlled and proceed in the ER [10]. Induced aggregation of misfolded or unfolded proteins in the ER eventually leads to BiP-mediated ER stress, which would induce the UPR to degrade these undesirable proteins [22]. As documented, IRE1*α*-, ATF6-, and PERK-mediated signaling transduction mechanisms are the main UPR pathways [23]. Of the three signaling pathways, emodin only induced the activation of IRE1*α*, which could increase the folding capacity of the ER by promoting the expression of XBP-1s [24]. At the same time, the entrance of misfolding or misfolding protein would be also repressed by the enhancement of IRE1*α*-XBP-1s signaling [25]. Hence, emodin induced the activation of IRE1*α*-XBP-1s signaling in order to restore the ER function. But on the other hand, a prolonged increase in IRE1*α*-XBP-1s signaling has been reported to trigger apoptosis via activating the downstream apoptotic factors, such as ASK1, p38 MAPK, and JNK [10]. Besides, CHOP, a multifunctional transcription factor, has been implicated in apoptosis in ER stress. Our study found that CHOP could be induced by IRE1*α*-XBP-1s rather than by PERK-ATF4 or PERK-eIF2*α* mediated the UPR, which has been reported to be stronger inducer signaling of CHOP [26, 27]. The expressions of downstream apoptotic genes of CHOP, for instance, *DOCs*, *GADD34*, and *TRB3*, and apoptosis ensued [28]. In short, the BiP/IRE1*α*/XBP-1s/CHOP signaling pathway might be involved in emodin-induced apoptosis and hepatotoxicity.

Previous studies have reported that CHOP could promote the expression of ER oxidoreduction 1*α* (ERO1*α*), which would redox regulate the activity of IP3R rather than PLC*γ* to cause Ca^2+^ release from the ER lumen [29]. Enhanced cytoplasmic Ca^2+^ content was likely to trigger the activation of calcium-dependent protein kinase II (CaMKII), which cleaved Caspase family members and finally activated various apoptotic pathways, including ER-related apoptosis that was mediated by Caspase-12 activation [30, 31]. Interestingly, enhanced CHOP-ERO1*α*-CaMKII activation could cause NADPH depletion and ROS generation in the ER lumen [32, 33]. As a result, emodin treatment might create a ROS-CHOP-positive feedback loop, which exacerbated oxidative injury induced by emodin. Also, we found that MT rescued emodin-induced mitochondrial function by inhibiting mitochondrial oxidative stress but could not regain intracellular Ca^2+^ homeostasis and suppress apoptosis, possibly because those ROS productions derived from mitochondria triggered excessive ROS generation in the ER lumen. Also, ROS in ER-caused Ca^2+^ leakage could not be canceled by reducing mitochondrially generated reactive oxygen species (mtROS) levels. Only when the release of Ca^2+^ from the ER to the cytoplasm was reduced could emodin-induced apoptosis be decreased. Hence, emodin exerted cytotoxic effects on human hepatocytes probably via ER-related apoptosis.

Recent advances have demonstrated the structural or mechanistic connections between the ER and mitochondria, for instance, mitochondrial-associated ER membranes (MAMs) [34]. Importantly, some of IP3Rs are located in MAMs and would provide a pathway to ER-mitochondrial Ca^2+^ transfers [35]. Therefore, emodin-induced ER stress and CHOP expression would enhance the ER Ca^2+^ influx to mitochondria, leading to mtROS in the mitochondrial matrix, both of which have been confirmed in emodin plus 2-APB-treated cells. Moreover, enough energy supply is necessary for protein folding and trafficking, the degradation of misfolded proteins in the ER [36]. Emodin-induced mtROS or Ca^2+^-induced secondary mtROS could both significantly inhibit mitochondrial oxidative phosphorylation. Also, Ca^2+^ overloading in the cytoplasm or mitochondria destroyed the normal Ca^2+^ gradient across the mitochondrial and ER membrane, which plays a key role in the mitochondrial ATP supply of the ER [37]. Altogether, emodin-induced oxidative inhabitation in mitochondrial function would aggravate ER-related apoptosis.

## 5. Conclusion

**Figure 7:**
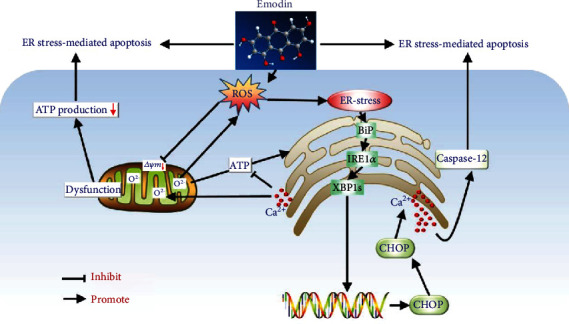
Schematic representation of the potential roles of oxidative stress mediated mitochondrial dysfunction and ER stress in emodin-induced hepatotoxicity.

In conclusion, as presented in Figure 7, we demonstrated for the first time that emodin caused obvious oxidative stress in human hepatocytes and ER-related apoptosis, which was associated with the activation of the BiP/IRE1*α*/CHOP signaling pathway and cytoplasmic Ca^2+^ overloading. During this progress, oxidative stress-mediated mitochondrial dysfunction would enhance emodin-induced-related hepatic apoptosis. Our findings are expected to help gain keen insights into toxic mechanisms underlying emodin-triggered hepatotoxicity and have implications for that oxidative stress-mediated ER stress could be an alternative target for the treatment of Cassiae Semen or other medicine-food homologous varieties containing emodin-induced liver injury.

## Data Availability

The data used to support the findings of this study are included within the article.

## Abbreviations

2-APB: 2-Aminoethyl diphenylborinate
CCK-8: Cell Counting Kit-8 assay
DCFH-DA: 2,7-dichlorofluorescein diacetate
DOX: Doxorubicin
ROS: Reactive oxygen species
Emo: Emodin
ER: Endoplasmic reticulum
MAMs: Mitochondrial-associated ER membranes
MMP: Mitochondrial membrane potential
MT: MitoTEMPO
mtROS: Mitochondrially generated reactive oxygen species
NAC: N-Acetyl-L-cysteine
OCRs: Oxygen Consumption Rates
TM: Tunicamycin
UPR: Unfolded protein response.

## Supplementary Materials

Supplementary Materials Table S1: information for all indicated antibodies used in the WB assay.
