# Supplementary material for: Emodin-Induced Oxidative Inhibition of Mitochondrial Function Assists BiP/IRE1α/CHOP Signaling-Mediated ER-Related Apoptosis
Source: Oxid Med Cell Longev. 2021 Apr 22;2021:8865813. doi: 10.1155/2021/8865813 (PMC8084644; doi:10.1155/2021/8865813)
Supplement: Supplementary Materials — Table S1: information for all indicated antibodies used in the WB assay. [file 8865813.f1.docx]

**Table S1 Information for all indicated antibodies used in WB assay.**

|  | **Name** | **Company** | **Dilution rate** |
| --- | --- | --- | --- |
| 1 | p-PLCγ | 530164, ZEN BIO, China | 1:1000 |
| 2 | p-PERK | 340846, ZEN BIO, China | 1:1000 |
| 3 | IRE1α | 3294, Cell Signaling Technology, USA | 1:1000 |
| 4 | Anti-Rb IgG | ab6721, Abcam, USA | 1:3000 |
| 4 | Anti-Ms IgG | ab6789, Abcam, USA | 1:3000 |
| 5 | ATF-6 | 65880, Cell Signaling Technology, USA | 1:1000 |
| 6 | ATF-6α | 500202, ZEN BIO, China | 1:1000 |
| 7 | BiP | 200310-4F11, ZEN BIO, China | 1:1000 |
| 8 | XBP-1s | 12782, Cell Signaling Technology, USA | 1:1000 |
| 10 | β-actin | 66009-1-Ig, Proteintech, USA | 1:1000 |
| 11 | p-eIF2α | 310073, ZEN BIO, China | 1:1000 |
| 12 | Caspase-12 | 2202, Cell Signaling Technology, USA | 1:1000 |
| 13 | CHOP | 15204-1-AP, Proteintech, USA | 1:1000 |
| 14 | Caspase-3 | 9662s, Cell Signaling Technology, USA | 1:1000 |
| 15 | Cytochrome c | 4280, Cell Signaling Technology, USA | 1:1000 |
| 14 | Bcl-2 | 4223T, Cell Signaling Technology, USA | 1:1000 |
| 15 | Bax | 5023S, Cell Signaling Technology, USA | 1:1000 |
